# Coarse-Grained Simulations Suggest Potential Competing Roles of Phosphoinositides and Amphipathic Helix Structures in Membrane Curvature Sensing of the AP180 N-Terminal Homology Domain

**DOI:** 10.1021/acs.jpcb.2c00239

**Published:** 2022-04-08

**Authors:** Alexis Belessiotis-Richards, Andreas H. Larsen, Stuart G. Higgins, Molly M. Stevens, Alfredo Alexander-Katz

**Affiliations:** † Department of Materials, Imperial College London, London SW7 2AZ, U.K.; ‡ Department of Bioengineering, Imperial College London, London SW7 2AZ, U.K.; § Institute of Biomedical Engineering, Imperial College London, London SW7 2AZ, U.K.; ∥ Department of Biochemistry, University of Oxford, Oxford OX1 3QU, U.K.; ⊥ Department of Materials Science & Engineering, Massachusetts Institute of Technology, Cambridge, Massachusetts 02139, United States

## Abstract

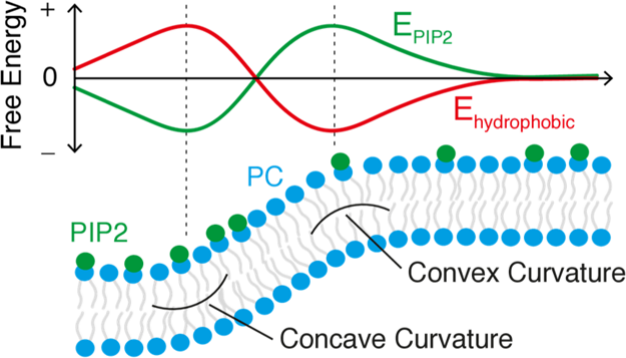

The generation and sensing of membrane curvature by proteins has become of increasing interest to researchers with multiple mechanisms, from hydrophobic insertion to protein crowding, being identified. However, the role of charged lipids in the membrane curvature-sensing process is still far from understood. Many proteins involved in endocytosis bind phosphatidylinositol 4,5-bisphosphate (PIP2) lipids, allowing these proteins to accumulate at regions of local curvature. Here, using coarse-grained molecular dynamics simulations, we study the curvature-sensing behavior of the ANTH domain, a protein crucial for endocytosis. We selected three ANTH crystal structures containing either an intact, split, or truncated terminal amphipathic helix. On neutral membranes, the ANTH domain has innate curvature-sensing ability. In the presence of PIP2, however, only the domain with an intact helix senses curvature. Our work sheds light on the role of PIP2 and its modulation of membrane curvature sensing by proteins.

## Introduction

Clathrin-assembly lymphoid myeloid leukemia (CALM) protein and its neuronal homolog AP180 contain clathrin and adaptor protein 2 (AP2) binding sites toward its C-terminus as well as an AP180 N-terminal homology (ANTH) domain that binds phosphatidylinositol 4,5-bisphosphate (PIP2) at its N-terminus.^1^ CALM binds PIP2 lipids in the cell membrane via its ANTH domain and mediates the assembly and disassembly of the clathrin coat during clathrin-mediated endocytosis (CME).^2−6^ The ANTH domain is also found in Sla2, a protein that is thought to mediate membrane–actin coupling.^7,8^ In addition, CALM is one of the many proteins recruited early on during CME and plays a role in stabilizing membrane curvature during their progression.^9,10^ Knockdown of CALM or AP180 disrupts endocytosis^11^ and synaptic vesicle formation.^12^

Miller et al. have shown that the CALM ANTH domain contains a terminal amphipathic helix (AH) that can induce membrane curvature and modulate the rate of endocytosis in cells.^13^ Such terminal amphipathic helices are thought to act as “wedges”, which insert into the membrane and drive membrane curvature.^14−16^ A key example of such a helix is found in the epsin N-terminal homology (ENTH) domain, another critical protein recruited early on during CME.^17^ Epsin is required to reconstitute clathrin-coated vesicles in vitro due to the curvature action of its AH and is critical for membrane fission.^18−20^ Recently, new research has begun to show the ability of these ENTH and ANTH domains to interact together and form complexes.^8,21−23^ As such, these protein domains and their terminal amphipathic helices play an important role in the initiation and progression of CME in cells. However, protein crowding and coupling with intrinsically disordered domains has also been noted as a mechanism for driving membrane curvature.^24−27^ It has even been shown that disruption of the key amphipathic helix (H0) in ENTH domains still allows for curvature generation due to crowding.^28^ In addition, our previous computational research has suggested that the ENTH domain can sense membrane curvature even without its terminal AH in the presence of PIP2.^29^ This contrasting evidence elicits questions regarding the role of AHs in membrane curvature sensing. Furthermore, both the ENTH and ANTH domains bind PIP2 lipids in the membrane but the role of these lipids surrounding curvature is still far from understood. Indeed, what role does PIP2 play with respect to curvature and localization of AH-containing proteins to membrane curvature? Also, how important are AHs in the curvature-sensing process?

**Figure 1:**
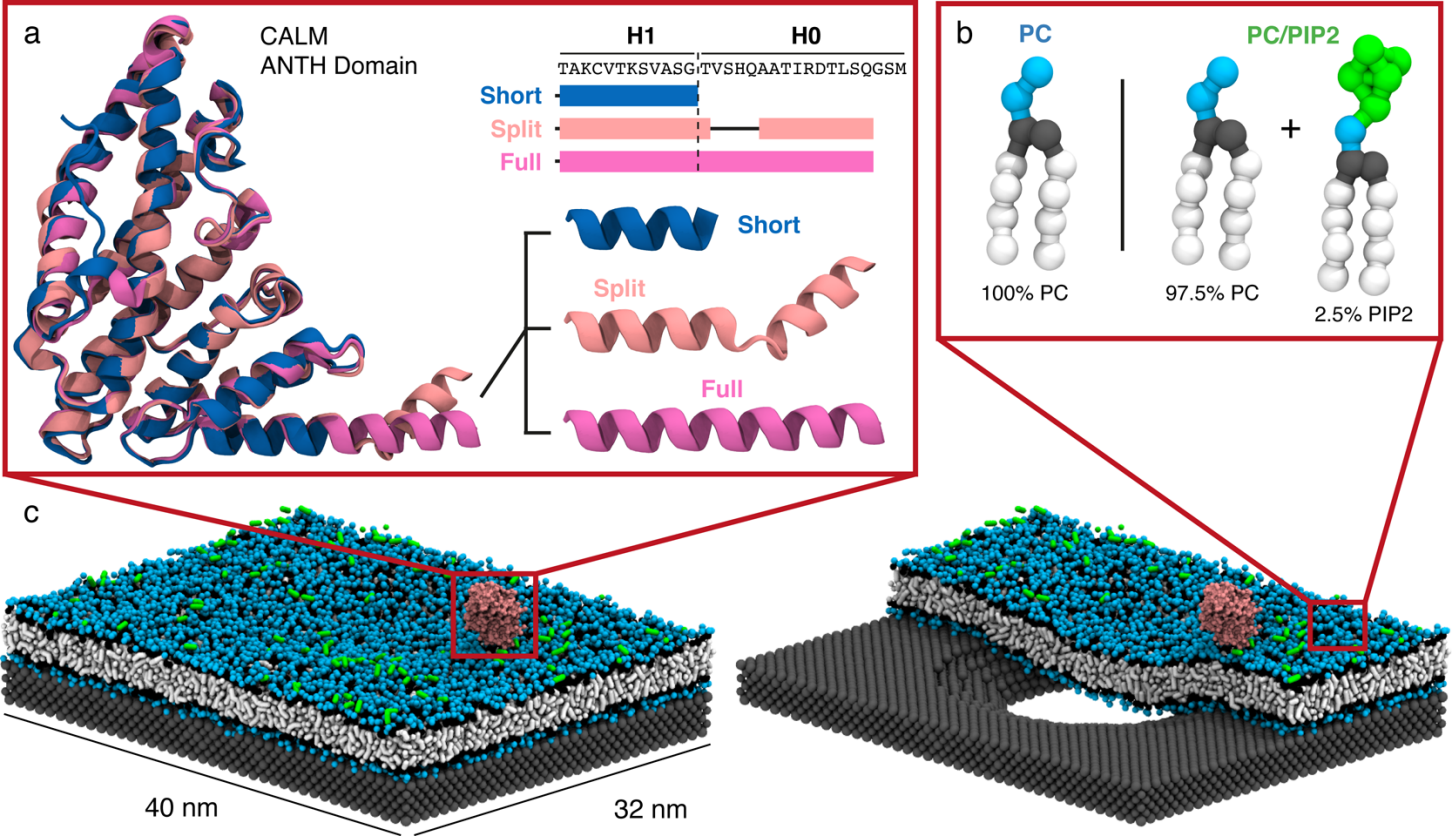
Schematic of the systems studied in this work showing (a) overlapped crystal structures of the CALM ANTH domain with the three terminal helices, denoted as: short (PDB code: 1HFA), split (PDB code: 3ZYM), and full (PDB code: 3ZYL), along with the amino acid sequence for this region. Note that each structure is missing the first three residues of H0 following Ford et al*.*^1^ (b) Snapshots of the lipids used in the two membrane compositions studied for this work, 100% PC (PC) and 97.5% PC + 2.5% PIP2 (PC/PIP2). White beads represent hydrophobic groups, gray beads represent glycerol groups, blue beads represent the headgroups of PC lipids, and green beads represent the headgroups of PIP2 lipids. (c) Snapshots of the simulation system showing the membrane as well as a slice-through highlighting curvature as well as a protein to-scale in red.

Following our previous works on protein–curvature interactions,^29,30^ we studied the behavior of the ANTH domain and its interaction with membrane curvature. We investigated the role of the ANTH’s terminal helix by evaluating the curvature-sensing ability of three crystal structures of the domain as proposed by Miller et al.^13^Figure 1a shows the crystal structure of the ANTH domain with three differing terminal helices, a short helix truncated at H1 (shown in blue), a split helix with an unstructured region separating H1 from H0 (shown in light pink), and a full helix with both H1 and H0 intact (shown in purple). In addition, we investigated how PIP2 affects the ANTH domain’s curvature-sensing behavior by studying two membrane compositions, a neutral 100% phosphatidylcholine (PC) membrane and a negatively charged membrane containing 97.5% PC and 2.5% PIP2 (see Figure 1b). Figure 1c shows snapshots of our simulation system showing a membrane interfaced with a nanoporous wafer as well as a slice through the membrane highlighting its curvature. Table 1 presents a summary of the simulations performed in this study.

**Table 1:** Summary of the Simulation Systems Investigated in This Work (* 97.5% PC and 2.5% PIP2)

simulation ID	protein	membrane	number of repeats	duration (μs)
short/PC	ANTH	PC	8	10
split/PC	ANTH	PC	8	10
full/PC	ANTH	PC	8	10
short/PC/PIP2	ANTH	PC/PIP2*	8	10
split/PC/PIP2	ANTH	PC/PIP2	8	10
full/PC/PIP2	ANTH	PC/PIP2	8	10

## Methods

### Simulation Details

All simulations were performed using Gromacs 2018.3.^31^ Protein simulations were performed at 323 K using the Martini 2 force field with explicit water.^32^ The temperature was coupled to a velocity rescale thermostat using a time constant of 1 ps with the protein, membrane, wafer, solvent, and ions each being coupled independently. The protein crystal structures were from the RCSB protein data base: ANTH short helix, [Protein Data Bank (PDB) code: 1HFA], ANTH with split helix (PDB code: 3ZYM), and ANTH with full helix (PDB code: 3ZYL). Proteins were coarse-grained using the *martinize.py*(33) script from cgmartini.nl, and an elastic network was added using a force constant of 500 kJ/mol and a cut-off distance of 0.9 nm. The systems were equilibrated for 20 ns using a 20 fs time step and a semi-isotropic Berendsen barostat with a time constant of 1 ps. For the production runs, a 30 fs time step was used with the semi-isotropic Parrinello–Rahman barostat with a 12 ps time constant. The protein center of mass was computed using the *gmx traj* tool, and contact analysis was performed using the *gmx mindist* tool, both in Gromacs.^31^ A velocity-rescaling thermostat was used throughout.

A rectangular simulation cell of dimensions 32 by 40 by 33 nm was used throughout. Proteins were placed 1.0 nm above membrane curvature, oriented with their PIP2 binding surface approximately facing the membrane, and located approximately at position (*x* = 2 nm, *y* = 16 nm, *z* = 15 nm) and run for 10 μs. Each protein/membrane combination was equilibrated and run right times to generate replicas from the starting position. Counter-ions were added to neutralize the system along with 15% antifreeze particles to avoid water freezing on the rigid substrate.^32^ The nanoporous wafer was constructed following our previous works.^29,30^ The wafer is built using Q0 beads, which are negatively charged to promote membrane adhesion.

To interface our membranes with the wafer, two 40 by 40 nm membranes containing either PC or PC and PIP2 were first built using the *insane* tool.^34^ Our membranes are built with dioleoyl (DOPC) tails for the PC lipids (known as DOPC in cgmartini.nl) and dioleoyl (DO-PIP2) tails for the PIP2 lipids (known as POP2 in cgmartini.nl). We only include PIP2 lipids in the top leaflet of the membrane as this is the region exposed to solvent and our proteins of interest. These were then sectioned into ribbons by removing 4 nm from either edge of the membrane along the *x*-direction, thereby leaving the membrane continuous only along *y*. These membranes were then equilibrated for 20 ns and simulated for 300 ns under the same simulation conditions mentioned previously. The equilibrated membranes were then placed above the nanoporous wafer and again equilibrated for 20 ns and simulated for 300 ns in order to interface with the wafer. We allow the membrane to interface with the wafer with free edges in order to promote lipid exchange and membrane remodeling at the free edges. This method ensures that we minimize any artificial tension introduced into the membrane during the curving process Once curved on the wafer, the unit cell was sliced from 40 by 40 nm to 32 by 40 nm in order to have a fully continuous membrane.

### Trajectory Analysis

All snapshots were taken using VMD.^35^ The heatmaps and radial distribution histograms were computed from the protein center of mass (CoM) data extracted from the simulations using the *gmx traj* tool mentioned above. Heatmaps in Figure 3 were created by converting the *x*–*y* CoM data into absolute distances from the central pore (*x*_c_ = 16 nm and *y*_c_ = 20 nm). This data was then mapped onto a 50 by 50 grid of equally spaced bins (*x* limits = 0–16 nm and *y* limits = 0–20 nm). The histograms (Figure 4) were determined by converting the *x*–*y* CoM data into the radial distance from the central pore following
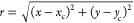
1where *r* is the radial distance of the protein and *x* and *y* are the spatial components of the protein CoM at a given frame. This radial data was then converted into a histogram over 100 equally spaced bins between 0 and 25 nm. In addition, kernel density estimates of the raw radial data were computed using the *stats.gaussian_kde* command from the python *scipy* package. Both the *x*–*y* histograms and radial histograms were normalized, so the sum over all bins in each histogram equals to 1.

To generate the data in Figures 5 and 6, we employed the *gmx mindist* Gromacs tool. Using this tool, we computed the average number of contacts between protein residues and either hydrophobic lipid tails (Figure 5) or PIP2 headgroups (Figure 6) over all the simulations performed for each protein structure studied.

### Curvature Evaluation

**Figure 2:**
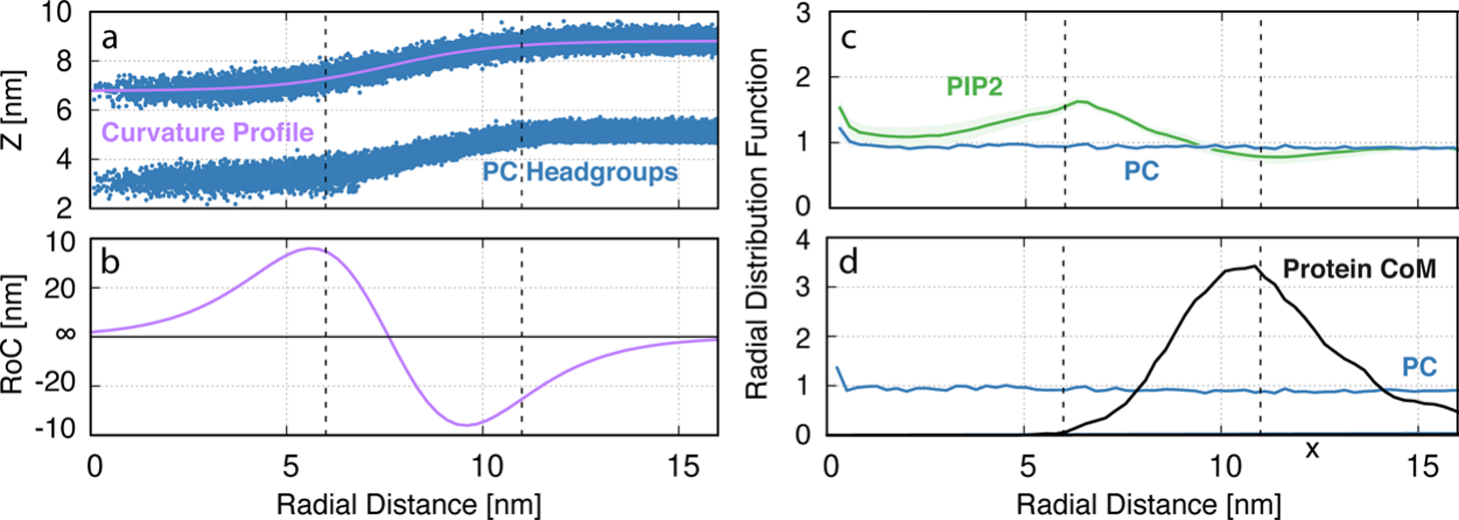
(a) Average vertical position of PC lipid headgroups and membrane curvature profile as a function of radial distance from central pore and (b) RoC values of curvature profile as a function of radial distance. (c) RDFs of PC and PIP2 lipids as a function of radial distance and (d) an example of how protein center of mass (CoM) localization is represented in this study. Note the dashed lines represent the start and end of the tapered region of the underlying wafer, which approximates to the membrane curvature region.

To quantify membrane curvature, we fit the curvature profile of the membrane, shown in Figure 2a, to the following sigmoid-like equation
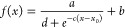
2where *a*, *b*, *c*, *d*, and *x*_0_ are fitting parameters. Once fitted, we can input the first and second derivatives of this function in order to calculate the radius of curvature (RoC) along the membrane
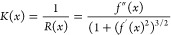
3where *K* is the curvature, *R* is the RoC, and *f*′(*x*) and *f*″(*x*) are the first and second derivatives of the sigmoid function fit of the membrane profile given, respectively, by
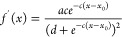
4
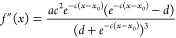
5

Equation 3 is used to plot the Roc along the membrane in Figure 2b.

## Results and Discussion

The curvature profile of our membrane system can be seen clearly in Figure 2a. The PC lipid headgroups are fitted to a sigmoidal function to get an estimate of the RoC (inverse of curvature) for our membrane (Figure 2b). In addition, due to the fixed curvature in our system, we observe PIP2 enrichment and depletion at concave (at approx. 6 nm) and convex (at approx. 11 nm) regions of the membrane, respectively (Figure 2c). In order to evaluate our protein curvature sensing, we compute the radial distribution functions (RDFs) of both protein and underlying lipid distributions, shown schematically in Figure 2d. These RDFs are normalized by the radial area as well as by each species’ global area density. As such, we can account for artifacts due to larger area effects and compare the local density of lipids and proteins with respect to their “bulk” density when their RDF equal to 1.

### Curvature Sensing of the ANTH Domain

**Figure 3:**
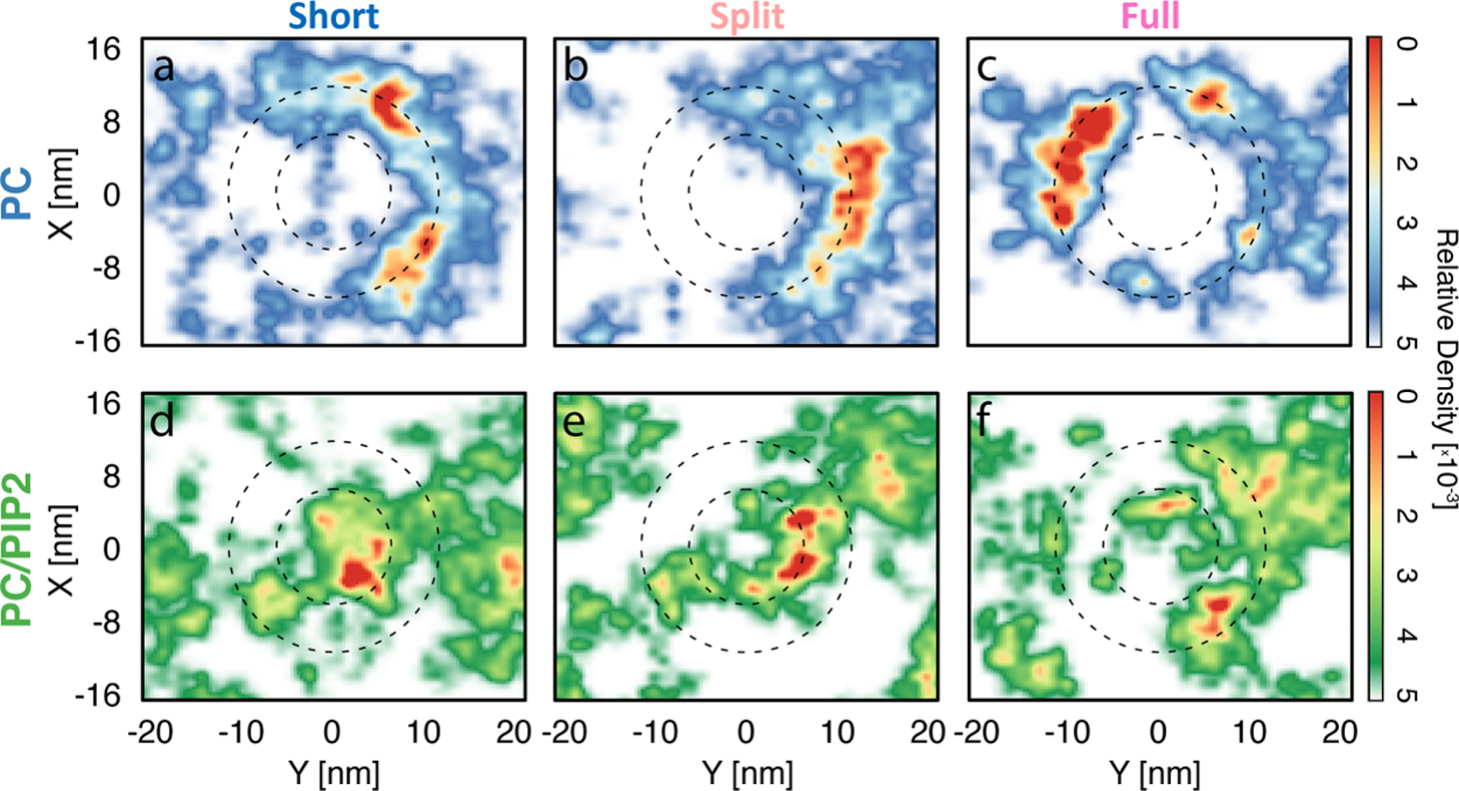
Top-down statistical frequency heatmaps showing the center of mass positions of (a,d) short, (b,e) split, and (c,f) full ANTH domains, respectively, on 100% PC and 2.5% PIP2 membranes across all simulations.

**Figure 4:**
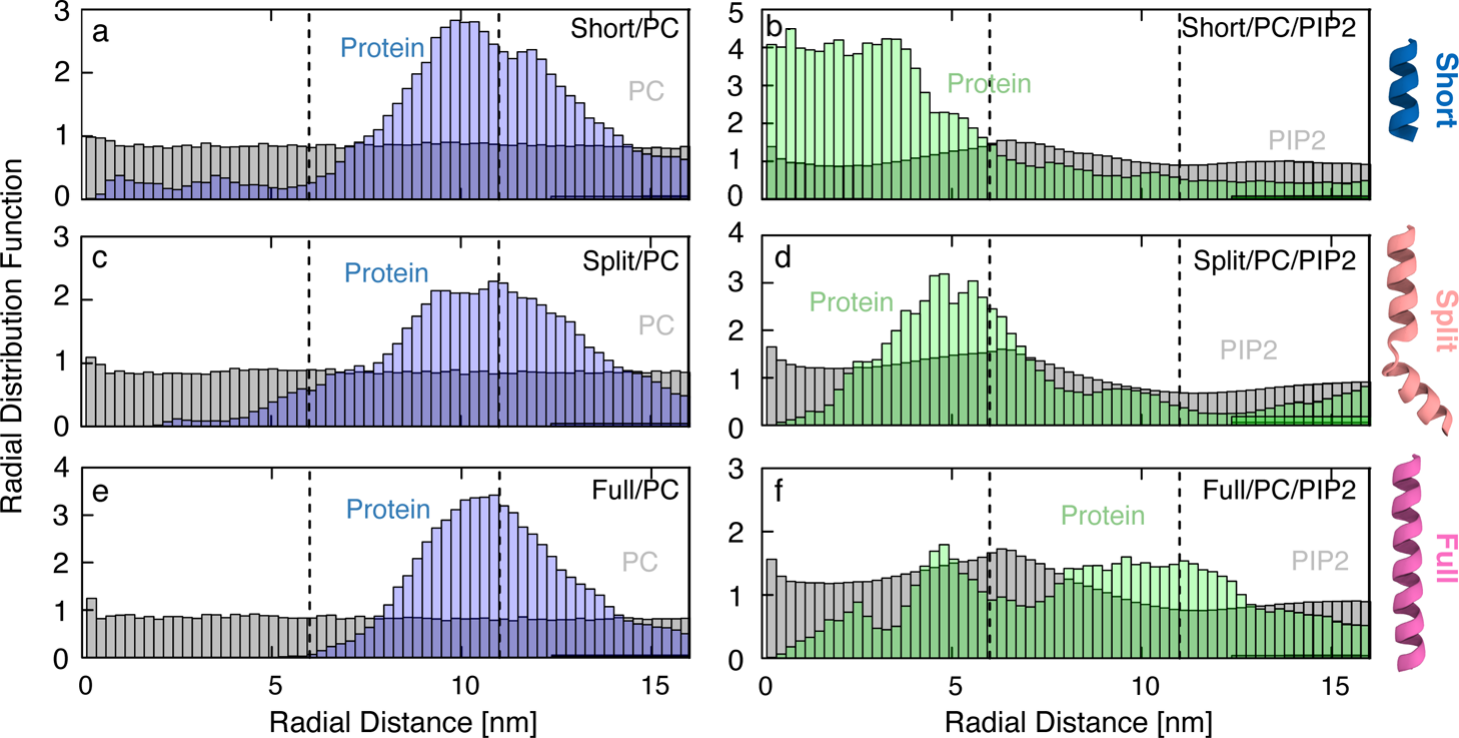
RDF of protein and lipid center of mass positions over 100 bins of (a,b) short, (c,d) split, and (e,f) full ANTH domains, respectively, on 100% PC and 2.5% PIP2 membranes. These RDFs are normalized by area and to each species’ global area density and highlight changes in local density with respect to the “bulk” density (RDF = 1). The dashed lines represent the start and end of the tapered region of the underlying wafer, which approximates to the membrane curvature region. Distributions are shown up to half the shortest box vector in *xy* (16 nm).

Examining the protein localization results shown in Figures 3 and 4, we can see consistent curvature sensitivity for all ANTH domains when interacting with 100% PC membranes (corresponding to peaks at approximately 11 nm in Figure 4a,c,e). This is surprising as, without a fully-fledged AH, we would expect both the short (Figures 3a and 4a) and Split (Figures 3b and 4c) ANTH domains to not be curvature active. These results suggest that the ANTH domain has some innate curvature sensitivity irrespective of the presence or structure of its AH. When PIP2 is added to the membrane, however, only the full ANTH domain retains its curvature-sensing ability (Figures 3f and 4f). Despite the strength of the full ANTH domain’s preference for curvature being weakened compared to the PC-only case (Figure 3e), it is the only protein structure simulated, which presents sensitivity to positive curvature in the presence of PIP2. This coincides closely with the experimental work performed by Miller et al., which suggests that only the full helix ANTH domain is curvature active.^13^ The Short/PC/PIP2 (Figure 4b) and Split/PC/PIP2 (Figure 4d) cases on the other hand show peaks in the concave region of the membrane (below 5 nm). This can be explained by PIP2’s preference for this region (Figure 2c) and as such PIP2 is driving the proteins spatial localization.

### Hydrophobic Interactions of the ANTH Domain

**Figure 5:**
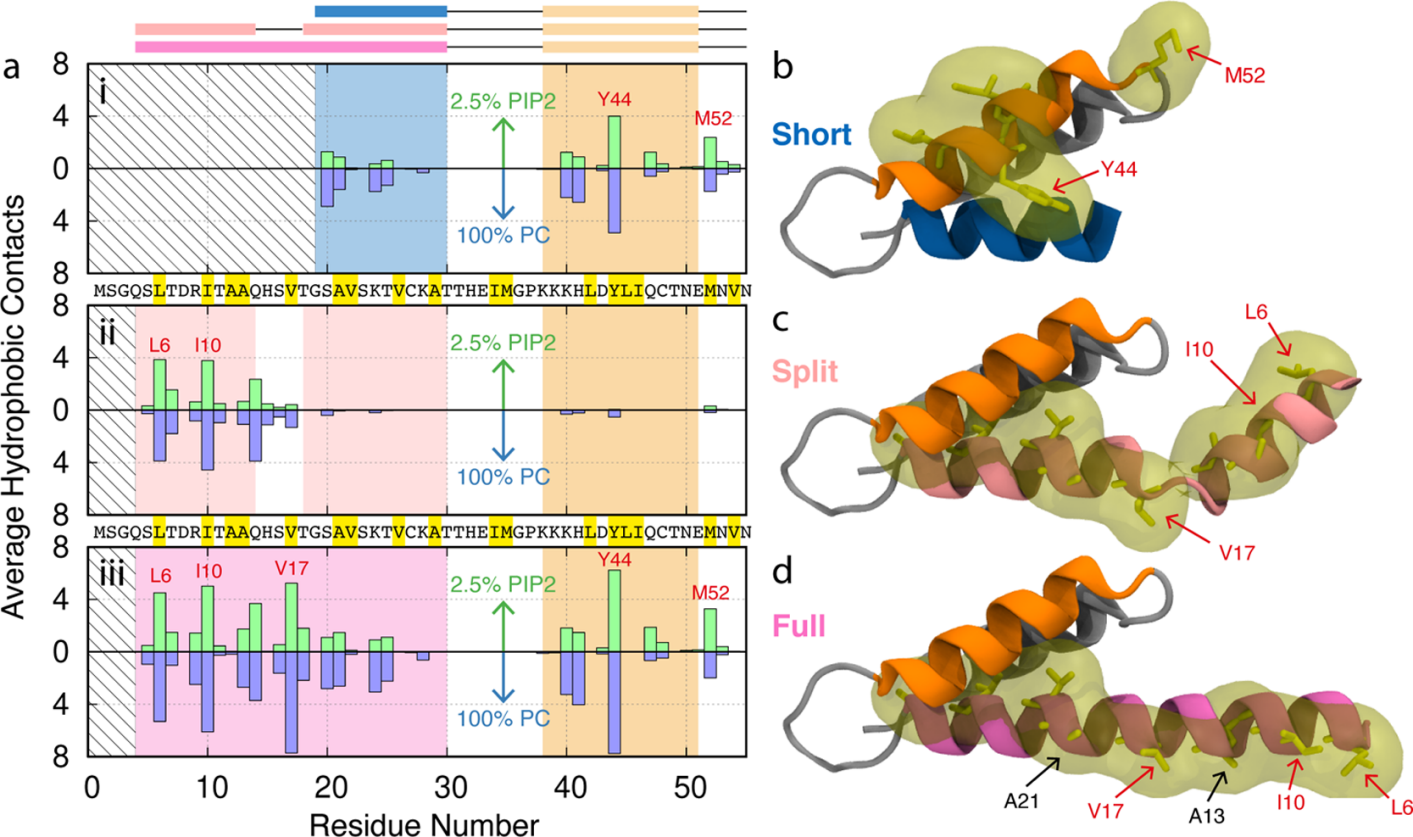
(a) Membrane binding analysis showing sequence and average hydrophobic contacts for (i) short, (ii) split, and (iii) full ANTH domains on 100% PC and 2.5% PIP2 membranes for the first 55 residues. Residue codes highlighted in yellow correspond to hydrophobic residues (L, I, A, V, M, and Y). The rectangles above (a) represent alpha helices in the structure. Residues L6, I10, V17, Y44, and M52 are marked in red text as they are the key hydrophobic residues in this region. Structures of first 55 residues of the (b) short (PDB code: 1HFA), (c) split (PDB code: 3ZYM), and (d) full (PDB code: 3ZYL) ANTH domains taken from experimental crystal structures. Hydrophobic residues and their corresponding hydrophobic surface are highlighted in yellow for residues 38–50 in (b) and for residues 0–30 in (c,d).

To understand the origin of the curvature sensing of the ANTH domain, we examined the binding behavior of our proteins to both PC and PC/PIP2 membranes. We analyzed the hydrophobic interactions between the protein and the hydrophobic tails of the lipid molecules in the membrane. Figure 5a shows the average hydrophobic contacts between each ANTH domain interacting with PC and PC/PIP2 membranes. For the short and full ANTH domains [Figure 5a(i,iii)], we see strong hydrophobic activity of two residues (Y44 and M52) on the third helix of the protein, denoted as H3. These residues can be seen on the structure of the ANTH domain in Figure 5b. The full ANTH domain [Figure 5a(iii)] also shows consistent hydrophobic activity along H0 and H1, with strong contacts at L6, I10, and V17, again corresponding very well with experimental work by Miller et al., which identified these as the key curvature-active residues of the ANTH domain.^13^ These three residues are highlighted and labeled in red on the structures in Figure 5c,d. The split domain, however, only shows strong activity at L6 and I10 [Figure 5a(ii)]. This binding information confirms that the ANTH domain’s curvature activity can be assigned to its AH. However, it also presents findings on how this protein interacts with curvature, with the hydrophobic activity of H3 being of interest. The hydrophobic binding information for all residues of the protein can be seen in Supporting Information, Figure S1a. Due to the fixed curvature of our system, we expect there to be more hydrophobic tail groups exposed to solvent at regions of negative curvature (around 11 nm), which will attract hydrophobic protein residues to bind there. In a non-fixed curvature setting, we can expect that the ANTH’s AH and hydrophobic residues will act as wedges to drive membrane curvature. It is also worth noting that, as opposed to the ENTH domain, we are unsure as to how the ANTH domain’s AH is formed. The ENTH domain’s AH forms as a structural consequence of binding PIP2.^17^ As we do not know whether PIP2 is necessary for the formation of the ANTH domain’s AH, we could posit that the Full AH of the domain impedes the binding of PIP2 lipids and hence allows the ANTH domain to sense curvature, ignoring the opposite pull of the PIP2 lipids in the central pore region. This is backed up by the fact that the Short helix ANTH domain can sense curvature without PIP2 suggesting that potentially all the hydrophobic residues on the Full helix may not be fully necessary for curvature sensing.

### PIP2 Binding of the ANTH Domain

**Figure 6:**
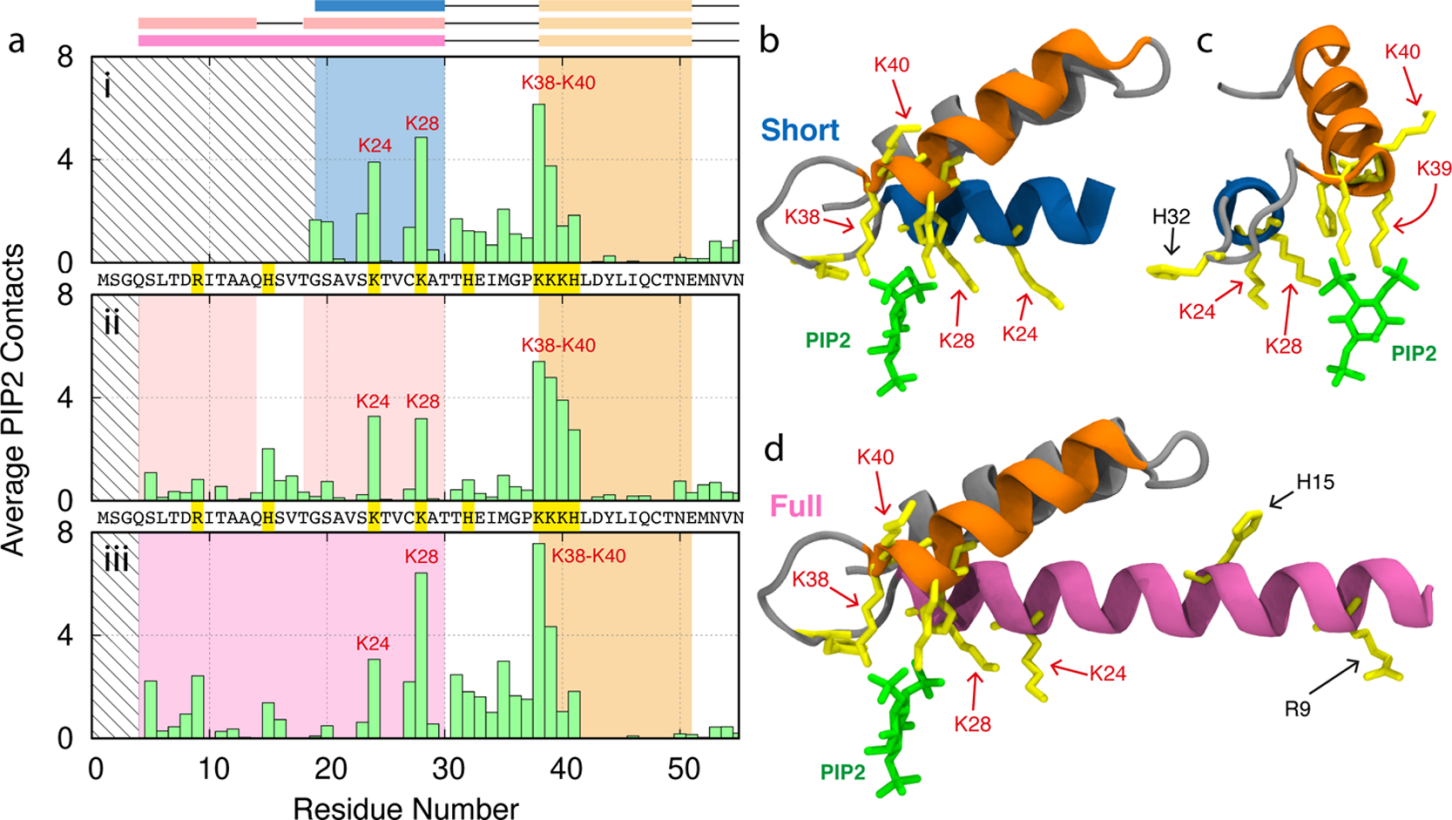
(a) Membrane binding analysis showing sequence and average PIP2 headgroup contacts for (i) short, (ii) split, and (iii) full ANTH domains on PC/PIP2 membranes for first 55 residues. Residue codes highlighted in yellow correspond to positively charged residues (K, H, and R). The rectangles above represent alpha helices in the structure. Residues K24, K28, and K38 to K40 are marked in red text as they are key PIP2-binding residues in this region. (b,c) Structure of first 55 residues of the short ANTH domain from the side and looking down H1, with PIP2 highlighted in green and positively charged residues highlighted in yellow. (d) Structure of first 55 residues of the full ANTH domain from the side. Note that location of PIP2 was determined by crystallography with the structure of the short helix (PDB code: 1HFA) reported by Ford et al.^1^ The position of PIP2 in (d) is purely representational.

After considering hydrophobic interactions, we examined the contacts between negatively charged PIP2 headgroups and the proteins. Figure 6a shows very consistent PIP2 binding across each case. Indeed, the key PIP2 binding residues from the literature are engaged (K28, K38, and K40)^1,13^ as well as another lysine residue (K24). These are highlighted in the structures in Figure 6b–d. This shows us that modifying the terminal helix of the ANTH domain does not affect PIP2 binding.

Supplementary Figure S1b shows the PIP2 binding for all residues of the ANTH domain. Interestingly, we see significant binding around residue 150. This can be explained by the ANTH domain tilting to maximize PIP2 contacts with other positively charged residues. Indeed, looking at the orientation of the ANTH domains on PC and PC/PIP2 membranes in Supporting Information, Figures S2 and S3, respectively, we see a clear orientation of the protein toward the membrane in the presence of PIP2, especially for the short and split cases. This was observed in a more quantitative fashion using rotation matrix calculations, where, in each case, we see a clear shift toward the membrane when PIP2 is added (Supporting Information, Figure S4).

### Summary of Results

**Figure 7:**
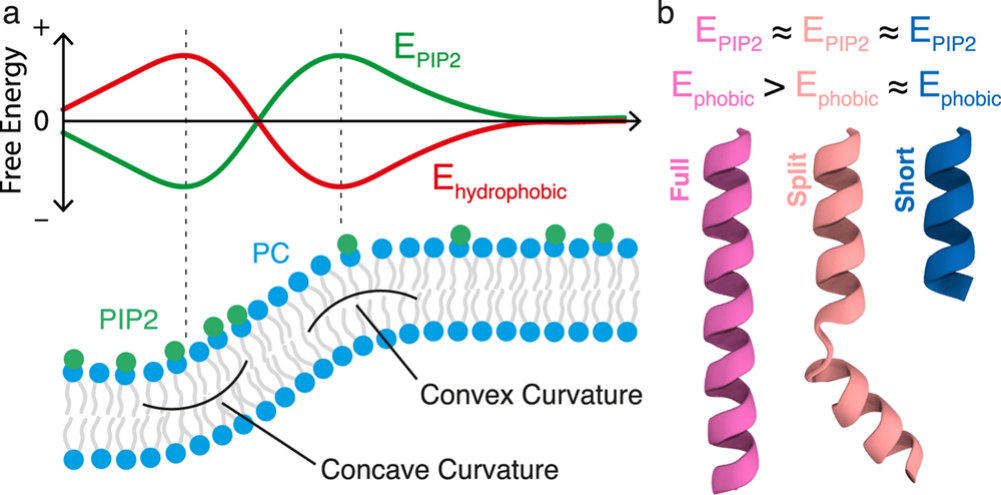
(a) Schematic of illustrated free energy profiles of PIP2 and hydrophobicity as a function of our membrane profile highlighting concave (∪) and convex (∩) regions of curvature. PIP2 shows preferential localization to concave curvature and as such its free energy contribution is negative at that region,^44^ while hydrophobic interactions are maximized at convex curvature. (b) Comparison of PIP2 and hydrophobic free energy contributions for full, split, and short helices of the ANTH domain along with their crystal structures.

Overall, our results show that despite having identical PIP2-binding sites, each structure of the ANTH domain senses curvature differently in the presence of PIP2 (Figures 3 and 4). We hypothesize that this behavior can be reconciled by considering the ANTH domain membrane localization behavior as a combination of the opposing PIP2- and hydrophobic-membrane interactions. While the contributions from PIP2 are identical for each crystal structure of the ANTH domain studied, the hydrophobic contributions for each helix structure are different (Figure 5). Indeed, the full helix shows five active hydrophobic residues (L6, I10, V17, Y44, and M52) and the split and short helices show only two each (L6, I10 and Y44, M52, respectively). This would suggest that the hydrophobic attraction to curvature for the short and split helices is lower than the full helix. Furthermore, from the localization behavior in Figures 3 and 4, we know that both the split and short helices cannot sense curvature in the presence of PIP2. PIP2 lipids are enriched away from convex curvature at flat and concave membrane regions. As such, these lipids are imposing an attractive force that is opposite to the hydrophobic attraction to convex curvature. Hence, we can rationalize that, in the short and split cases, PIP2 attraction is stronger than hydrophobic attraction leading to a loss of curvature sensing (Figure 4b,d). The full helix, however, has a sufficiently strong hydrophobic contribution that it can overcome this opposing PIP2 attraction and still sense curvature (Figure 4f). This logic is represented schematically in Figure 7. Figure 7a shows a schematic of illustrated free energy contributions from PIP2 and hydrophobicity along our curvature model, while Figure 7b shows the relative contributions of each opposing forces for each full, split, and short helix.

Two strong limitations of our curvature methods stand out. First, we employ solely production molecular dynamics simulations in order to validate equilibrium binding and localization behavior of the ANTH domain on membranes. More robust free-energy methods would improve the conclusions of our current work. Despite attempts, our system size and computational resources have not been adequate to supplement our results with converged umbrella sampling or metadynamics results. Furthermore, it should be noted that our substrate-induced curvature model is far from ideal. Although the artifacts and disruptions to membrane behavior are minimal, this model lacks both elegance and adaptability. Indeed, whole new substrates need to be built and designed to accommodate different curvature radii and only one side of the membrane is accessible to solvent and proteins. It should be noted that research groups studying similar curvature processes have found success using buckled membrane models to control curvature.^36,37^ While our model does allow for some control over the scale of the curvature introduced into the membrane, future works could make use of the new EnCurv tool developed by Yesylevskyy and Khandelia to gain use of the adaptable membrane curvature without the need for rigid substrates.^38^ In addition, one should note that the Martini model has recently been scrutinized for the excessive “stickiness” by the developers, in particular regarding protein–protein interactions.^39^ Although there is no direct evidence to our knowledge of excessive stickiness regarding protein–lipid interactions, future work employing atomistic models or competing coarse-grained models would be welcome to validate our observed protein behavior. This “stickiness” could potentially contribute to the PIP2’s role in dampening the curvature sensitivity of the Short and Split ANTH domains.

It is interesting to note the concave curvature preference of PIP2. Due to the lipid’s bulky and charged headgroup, it would seem natural for PIP2 to prefer convex curvature.^40^ However, Tsai et al. have shown that no curvature-induced sorting of PIP2 occurs in membrane tubules,^41^ nor was PIP2 preference for membrane curvature observed in Zhao *et al.*’s study of nanobar-deformed membranes,^42^ and simulation studies imply that this geometrical effect is modest.^43^ Coarse-grained MD simulation studies have, on the other hand, shown the opposite effect, namely PIP2 clustering at concave membrane curvature.^44−46^ Although this concave preference is consistent with the forcefield used in this study, more work is needed to explore the curvature localization of PIP2 molecules. Atomistic simulations, and experiments in particular, are needed to validate these coarse-grained predictions of PIP2 localization to the concave curvature.

## Conclusions

In conclusion, we have studied the membrane curvature activity of the ANTH domain with three different helix structures under PC-only and PIP2-containing membrane conditions. We have shown that on PC-only membranes, the ANTH domain exhibits curvature-sensing behavior irrespective of the structure of its terminal helix. This is surprising as we expect this helix to be the main driver behind curvature sensing of the ANTH domain. This can be explained by the activity of two hydrophobic residues on helix H3, Y44 and M52, at the membrane-exposed surface of the protein. On PIP2-containing membranes, only the ANTH domain with a full helix can sense curvature, while the other ANTH domain variants localize to PIP2 dense regions. This can be explained by the combined activity of hydrophobic residues on both helices H0 and H3. Our hydrophobic binding analysis for H0 ratified the experimental results suggesting that residues L6, I10, and V17 are the key curvature active residues on this helix. The short and split cases, on the other hand, have less overall hydrophobic residues exposed and as such do not sense membrane curvature. Finally, we showed that all three protein structures bind PIP2 in the same manner (matching experimental work).

Overall, this work sheds light on the complex interaction between PIP2-binding proteins and curvature. In essence, we believe that there is a balance of opposite forces due to the PIP2 localization away from regions of convex curvature to the central and flat regions of the membrane. We can extrapolate this result to suggest a more generalized mechanism for curvature-sensing proteins during endocytosis. Due to the local enrichment of PIP2 during endocytosis, we can imagine that most of these lipids will localize around the forming bud, at concave curvature. Hence, curvature-active proteins must be able to balance their hydrophobic and PIP2 attractions in order to bind PIP2 at endocytic pits and still localize to curvature in order to drive the CME process. One could conjecture that this exquisite control over the localization of the proteins can be useful to first localize PIP2-associated proteins and then disperse them in the more defined nascent endosome as curvature increases during the endocytosis process. Future work could mimic this by exploring lipid and protein localization to varying membrane curvature levels.

## Supporting Information Available

Simulation details and details on the simulation system used; protein localization behavior to curved membranes; and protein binding behavior to both curved membranes (PDF)

## Abbreviations

PIP: phosphatidylinositol phosphate
ANTH: AP180 N-terminal homology
DOPC: dioleoylphosphatidylcholine
CME: clathrin-mediated endocytosis
