# Supplementary material for: Coarse-Grained Simulations Suggest Potential Competing Roles of Phosphoinositides and Amphipathic Helix Structures in Membrane Curvature Sensing of the AP180 N-Terminal Homology Domain
Source: J Phys Chem B. 2022 Apr 8;126(15):2789–97. doi: 10.1021/acs.jpcb.2c00239 (PMC9036517; doi:10.1021/acs.jpcb.2c00239)
Supplement: Supplementary file 1 — jp2c00239_si_001.pdf [file jp2c00239_si_001.pdf]

Supplementary Information

Coarse-Grained Simulations Suggest Potential Competing Roles of Phosphoinositides and Amphipathic Helix Structure in Membrane Curvature Sensing of the AP180 N-Terminal Homology Domain

Alexis Belessiotis-Richards,[1,2,3] Andreas H. Larsen,[4] Stuart G. Higgins,[1,2,3] Molly M. Stevens[1,2,3*] and Alfredo Alexander-Katz[5*]

[1]Department of Materials, Imperial College London, London SW7 2AZ, United Kingdom

[2]Department of Bioengineering, Imperial College London, London SW7 2AZ, United Kingdom

[3]Institute of Biomedical Engineering, Imperial College London, London SW7 2AZ, United Kingdom

[4]Department of Biochemistry, University of Oxford, Oxford OX1 3QU, United Kingdom

[5]Department of Materials Science & Engineering, Massachusetts Institute of Technology, Cambridge, MA 02139, USA

*Corresponding Authors

E-mails: m.stevens@imperial.ac.uk (M.M.S.); aalexand@mit.edu (A.A.K.)

Supplementary Figures

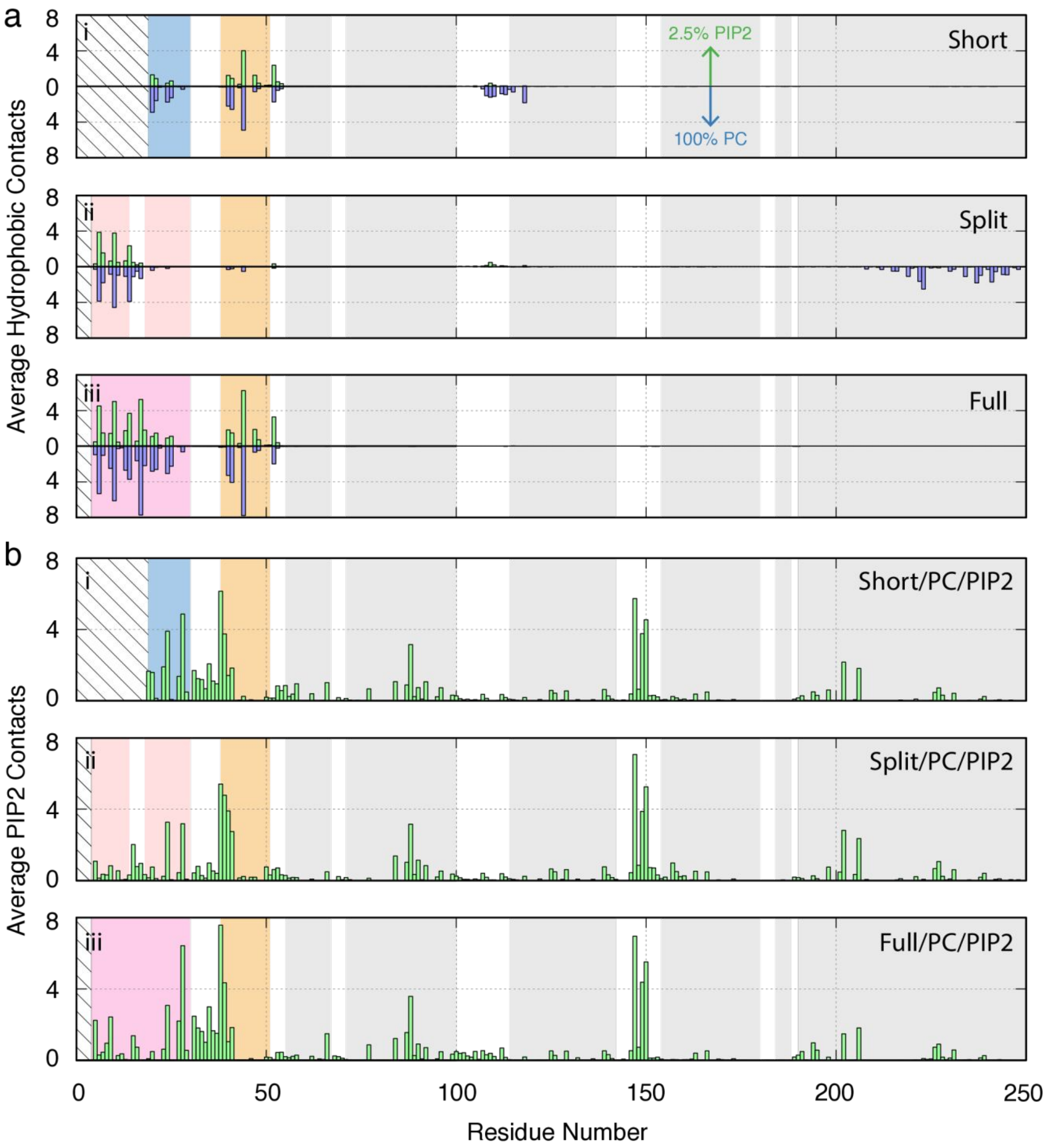

Figure S1: (a) Binding information showing hydrophobic contacts for (i) Short, (ii) Split and (iii) Full cases on both PC and PC/PIP2 membranes. (b) Binding information showing PIP2 contacts for (i) Short/PC/PIP2, (ii) Split/PC/PIP2 and (iii) Full/PC/PIP2 cases. Note the colored highlights in the plot represent the locations of alpha helices on the protein.

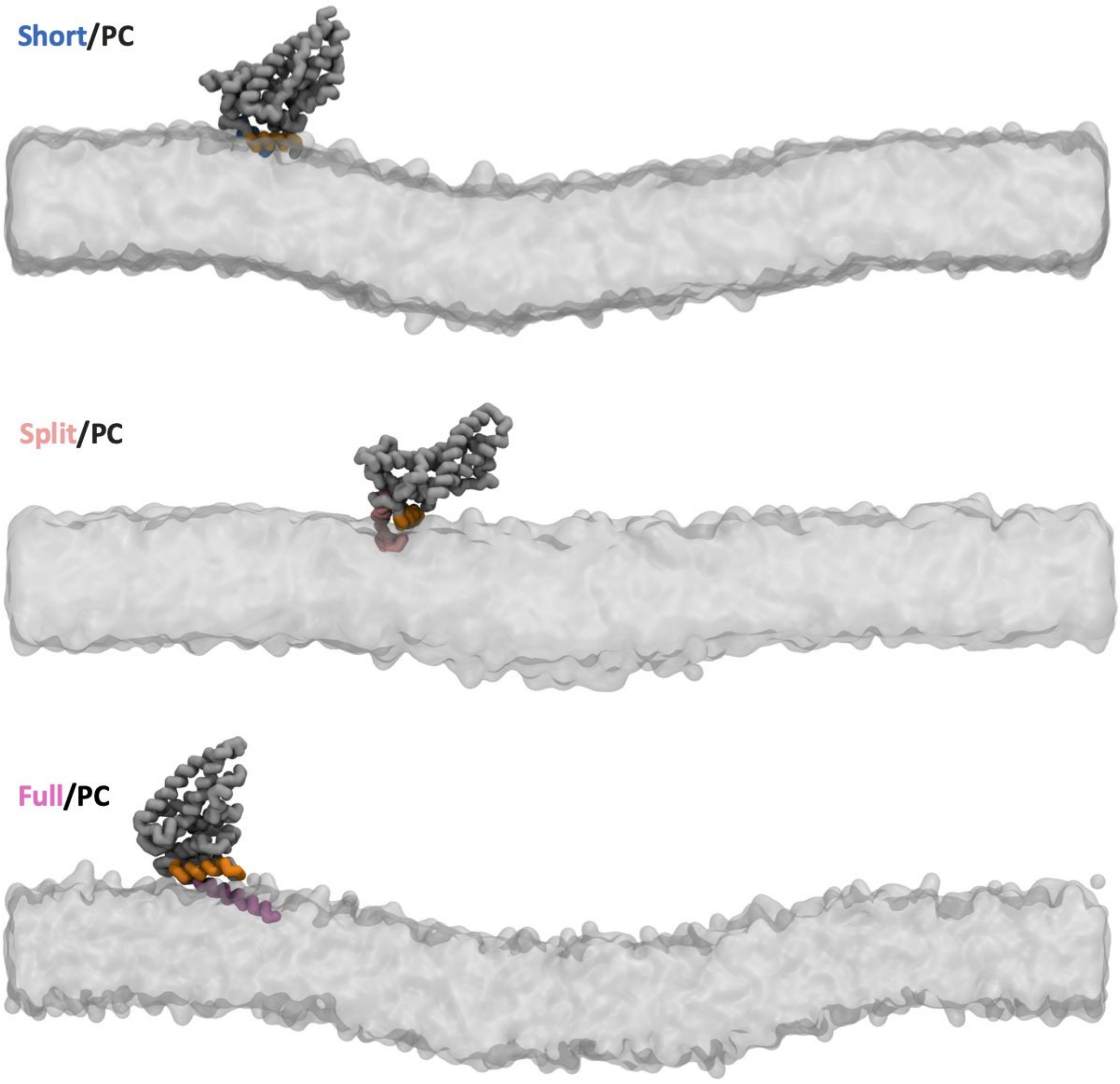

Figure S2: Snapshots of the ANTH domain interacting with PC-only membranes.

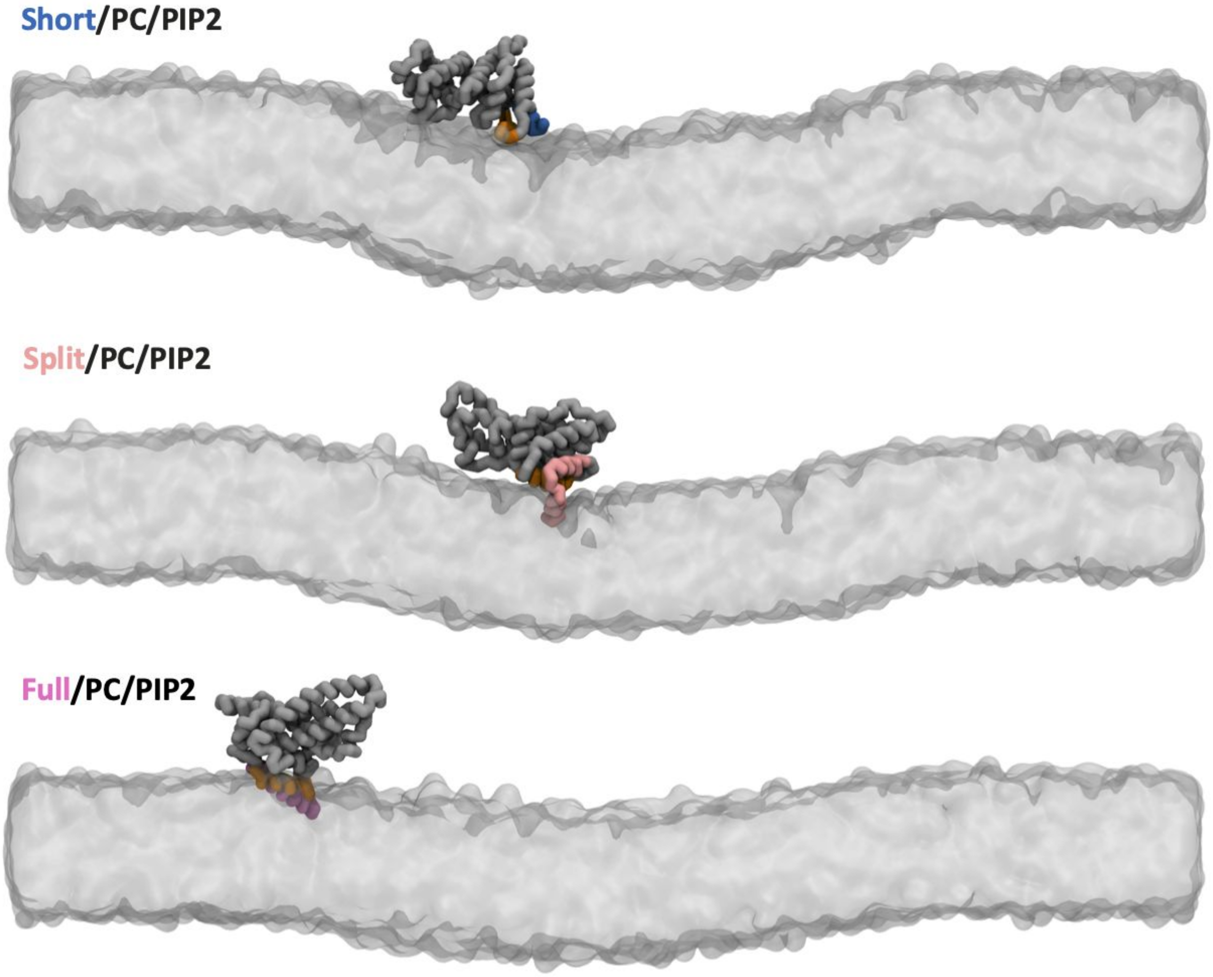

Figure S3: Snapshots of the ANTH domain interacting with PIP2-containing membranes.

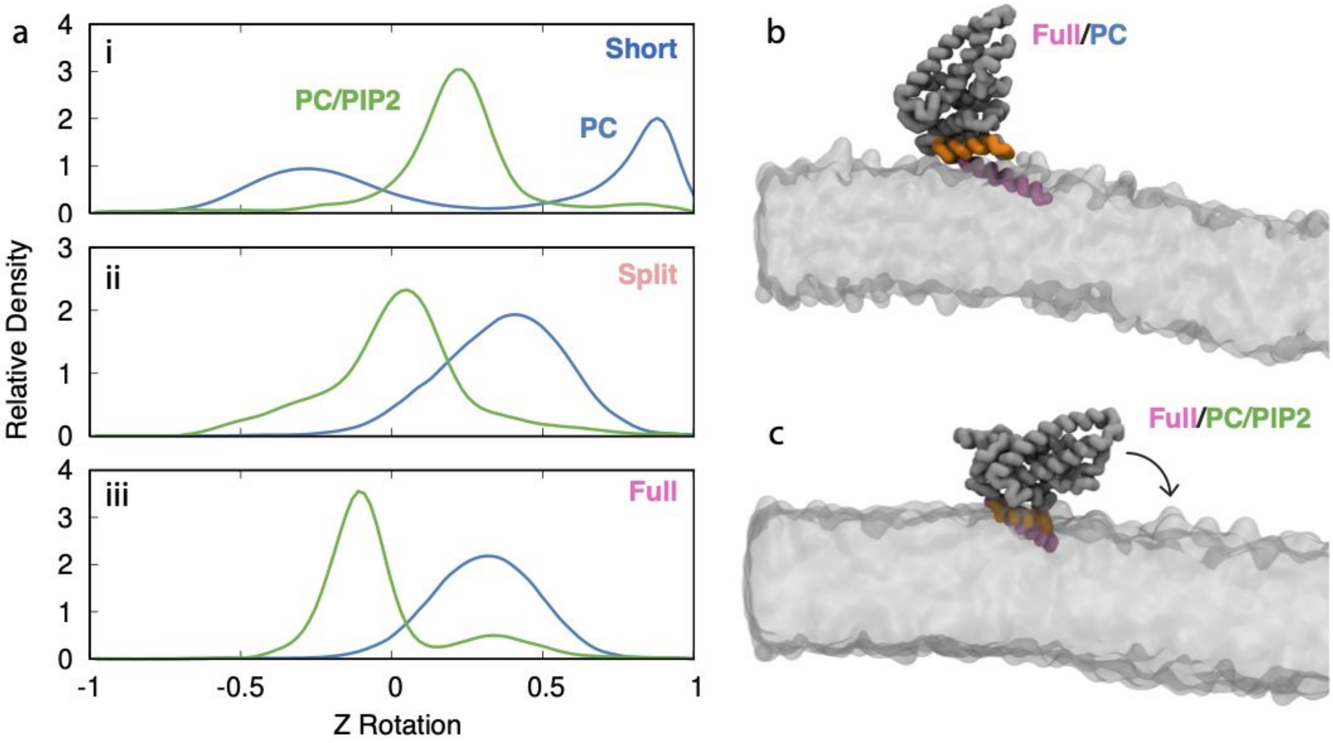

Figure S4: (a) Relative density of z component rotation matrix compared to initial state for (i) Short, (ii) Split and (iii) Full helix cases on both PC and PC/PIP2 membranes. (b) and (c) show snapshots of the Full ANTH domain interacting with a PC and PC/PIP2 membrane respectively showing tilt of the protein orientation once bound in the PIP2-containing case.
